# Draft Genome Sequence of *Rhodococcus qingshengii* Strain BKS 20-40

**DOI:** 10.1128/genomeA.00128-13

**Published:** 2013-03-28

**Authors:** Monu Bala, Shailesh Kumar, Gajendra Pal Singh Raghava, Shanmugam Mayilraj

**Affiliations:** a Microbial Type Culture Collection and Gene Bank (MTCC), Chandigarh, India; b Bioinformatics Centre, CSIR-Institute of Microbial Technology, Chandigarh, India

## Abstract

We report the 5.8-Mb genome sequence of *Rhodococcus qingshengii* strain BKS 20-40, isolated from a palm tree rhizosphere soil sample from Bhitarkanika National Park, Odisha, India. The strain is capable of degrading cholesterol moiety. The draft genome of strain BKS 20-40 consists of 6,601,618 bp, with 62.4% G+C content.

## GENOME ANNOUNCEMENT

The genus *Rhodococcus* was first proposed by Zopf (1891) and emended by Tsukamura (1974) (1) and Goodfellow and Alderson (1977) (2). *Rhodococcus qingshengii* was first characterized by Jing-Liang Xu et al. (3). *R. qingshengii* strain BKS 20-40 is a Gram-positive, aerobic, nonmotile, mesophilic strain, isolated from a palm tree rhizosphere soil sample collected from the Bhitarkanika Mangrove Reserve Forest, Odisha, India. During screening, we found that this strain is capable of producing cholesterol oxidase. The genome of *R. qingshengii* BKS 20-40 was sequenced using the Illumina-HiSeq 1000 paired-end technology, which produced a total of 28,206,978 paired-end reads (insert size of 350 bp) of 101 bp. We have used the next-generation sequencing quality-control NGS QC toolkit v2.3 (4) to filter the data for high-quality (HQ) (cutoff read length for HQ = 70%; cutoff quality score = 20) vector- and adaptor-free reads for genome assembly. A total of 26,791,554 high-quality vector-filtered reads (~541.2× coverage) were used for assembly with Velvet 1.2.08 (at a hash length of 55) (5). The final assembly contains 104 contigs of total size 6,601,618 bp, with an N_50_ contig length of 175.3 kb; the largest contigs assembled measure 483.3 kb.

The draft genome (104 contigs) comprising 6,601,618 bp was annotated with the help of the Rapid Annotations using Subsystems Technology (RAST) (6) and RNAmmer 1.2 (7) servers. A total of 6,409 predicted coding regions (CDSs), 3 rRNAs, and 52 tRNAs were predicted.

RAST annotation indicates that strain *Rhodococcus jostii* RHA1 (score 511), *Rhodococcus erythropolis* PR4 (score 494), and *Rhodococcus erythropolis* SK121 are the closest neighbors of strain BKS 20-40. RAST server annotation also indicates that strain BKS 20-40 has the genes coding for glycolysis, gluconeogenesis, and the tricarboxylic acid (TCA) cycle. Strain BKS 20-40 also has the genes encoding the acetyl-coenzyme A (CoA) fermentation to butyrate, including enoyl-CoA hydratase (EC 4.2.1.17), acetyl-CoA acetyltransferase (EC 2.3.1.9), butyryl-CoA dehydrogenase (EC 1.3.99.2), 3-hydroxybutyryl-CoA epimerase (EC 5.1.2.3), 3-hydroxybutyryl-CoA dehydrogenase (EC 1.1.1.157), 3-hydroxyacyl-CoA dehydrogenase (EC 1.1.1.35), and electron transfer flavoprotein (both alpha and beta subunits). In the annotation, we have also found the genes encoding 3-ketoacyl-CoA thiolase (EC 2.3.1.16), long-chain-fatty-acid-CoA ligase (EC 6.2.1.3), and aldehyde dehydrogenase (EC 1.2.1.3).

### Nucleotide sequence accession numbers.

The draft genome sequence of *R. qingshengii* BKS 20-40 has been included in the GenBank Whole-Genome Shotgun (WGS) database under the accession no. AODN00000000. The version described in this paper is the first version, accession no. AODN01000000.
